# Research on the Similarity Law of the Fragmentation Effect of a Reactive Inner Core PELE Penetrating a Steel Plate

**DOI:** 10.3390/polym18131590

**Published:** 2026-06-26

**Authors:** Yongjin Lu, Bo Tan, Shixi Yang, Shiyan Sun, Gangwei Liu, Da Deng

**Affiliations:** 1 Naval University of Engineering, Wuhan 430033, China; d22382602@nue.edu.cn (Y.L.);; 2 Xi’an Modern Chemistry Research Institute, Xi’an 710065, China

**Keywords:** PTFE/Al material, reactive inner core PELE, similarity, deflagration reaction, dispersion radius

## Abstract

This study explores the similarity of the fragmentation effect of a reactive inner core PELE (RIC-PELE) when penetrating a steel plate by measuring the broken length of the jacket after perforating the steel plate and the dispersion radius of the jacket fragments behind the steel plate. Based on the dimensional theory, the dimensionless functions of these two physical quantities were analyzed and established. On the basis of verifying the validity of numerical simulation, the penetration and deflagration damage effects of five scale models were simulated on the ANSYS/Autodyn 17.0 software platform, and the dimensional analysis was verified. In the obtained dimensionless functions, the independent variables are all geometric dimensionless quantities. The simulation results reveal that, within the impact velocity range of 900–1900 m/s, the dimensionless broken length of the jacket and the dimensionless dispersion radius of jacket fragments behind the target are approximately equal in different scale models at the same velocity; these values fall within error margins of ±7% and ±9% of the reference model, respectively, and both dimensionless quantities exhibit an approximately linear positive relationship with impact velocity. This indicates that when ignoring the size effect caused by the strain rate effect of the materials, the geometric similarity law of the fragmentation effect of a RIC-PELE penetrating a steel plate essentially holds, thereby verifying the correctness of the dimensional analysis.

## 1. Introduction

A penetrator with enhanced lateral effect (PELE) is a new type of kinetic energy projectile proposed by Kerk [1] and Paulus et al. [2]. The traditional PELE is mainly composed of a jacket and an inert inner core. The jacket material is usually a high-density metal such as tungsten and steel, which have excellent penetration performance; in contrast, the inner core is made from low-density materials such as aluminum and polyethylene, which have relatively weak penetration ability [3]. This kind of munition is equipped with neither explosives nor fuses. During the interaction with the target, the high-density, high-strength jacket penetrates the target plate, while the low-density, low-strength core is subjected to intense axial compression, resulting in an increase in the jacket’s internal pressure. According to the Poisson effect, the accumulated axial compression energy during the penetration process is converted into radial expansion energy. Consequently, the inner core exerts a radial force on the jacket. When the PELE perforates the target plate, due to stress unloading and energy release, the jacket breaks into a large number of radially scattered fragments, forming a post-target fragment killing field.

To date, researchers have conducted numerous studies on the lateral enhancement effect of conventional PELEs and their influencing factors. Paulus and Schirm [4] experimentally investigated the expansion fracture and radial scatter of the jackets of PELEs with different core materials—namely, aluminum and polyethylene—after perforating the target. Furthermore, the influence of the impact velocity of the penetrator, along with the thickness and material of the target plate, was also studied. Based on the principle of energy conservation and the one-dimensional linear elastic wave theory, Paulus and Schirm [4] established theoretical models for the axial residual velocity of the PELE and the radial scattering velocity of the fragments behind the target. However, this model underestimates the pressure of the shock wave; therefore, it is only effective for weak impacts. According to the principle of energy conservation and the theory of shock waves, Verreault et al. [5,6,7] established a theoretical model of the radial scattering velocity of PELE fragments, considering the interaction between the jacket and the inner core. Fan et al. [8] utilized shock wave theory to establish a two-stage analytical model, which is more accurate than the calculation results of the elastic wave theory. Cheng et al. [9] studied the influence laws of impact velocity and target plate material on the dispersion radius of fragments of a 12.7 mm caliber radially layered PELE through experiments and numerical simulation. In addition, Cheng et al. [10] carried out damage tests on multi-layer interval metal targets using three types of PELEs with a diameter of 25 mm (single-layered tungsten alloy jacket, radially layered tungsten alloy jacket, and radially layered W/Zr-based amorphous composite jacket), and the damage mechanism and the degree of damage dealt to each target plate by the three types of penetrators were analyzed in detail.

In the field of large-caliber PELEs, Lei et al. [11] demonstrated that the interaction behavior of shock waves and rarefaction waves, which varies with the inner-to-outer diameter ratio of the jacket, serves as an important mechanism leading to different lateral effects in different configurations of the penetrators. By studying the penetration of large-caliber PELEs into reinforced concrete targets, Xu et al. [12] proposed an analytical model to describe the deformation pattern of the jacket during the penetration process and established a relational equation between the perforation diameter of the target plate and the diameter of the penetrator. They pointed out that when the impact velocity and the strength and thickness of the target plate remain unchanged, the strength and thickness of the jacket affect the deformation mode of the jacket by influencing the bending resistance performance, thereby affecting the perforation diameter, while the strength of the core material indirectly affects the perforation diameter by changing its own expansion and failure. Furthermore, two dimensionless parameters were determined and a dimensionless perforation diameter model was established [13,14]. Based on dimensional theory, Xu et al. [15] performed a similarity analysis on the broken length of the PELE jacket and the dispersion radius of the jacket fragments after perforating the metal target. The results demonstrated that the fragmentation effect of PELEs conforms to a strict geometric similarity law, and the validity of the theoretical analysis was further corroborated through experiments and numerical simulations.

From these studies, it is evident that the research on the projectile–target interaction mechanism, the axial residual velocity model, the radial scattering velocity model of jacket fragments of traditional PELEs, and the damage mechanism and influencing factors is relatively sufficient, while the similarity research on PELE penetration damage is comparatively lacking. However, these studies have provided substantial theoretical foundations for research related to PELEs with new reactive inner core materials.

PTFE/Al is a reactive material that exhibits characteristics of low density, low strength, and a relatively high Poisson’s ratio, making it well suited as a core material for PELEs. In comparison with conventional PELEs, those incorporating PTFE/Al as their inner core material (i.e., reactive inner core PELE, denoted as RIC-PELE) demonstrate the following distinct advantages: PTFE/Al represents a unique class of energetic material that maintains stability under normal conditions [16,17], but when subjected to high-velocity impact or high-strain-rate loading, it undergoes deflagration [18,19,20,21,22] or an impact-induced detonation reaction [23,24,25], releasing substantial chemical energy, which significantly enhances the radial scattering velocity of the jacket fragments behind the target [26]. Zhou et al. [27] showed that the jacket fragments of RIC-PELEs with a truncated conical head can increase the range of damage to the rear target by 43% compared with the traditional PELE. In accordance with the principle of energy conservation, Ding et al. [28] systematically analyzed the energy sources contributing to the radial scattering velocity of RIC-PELE jacket fragments behind the target, which include (1) the axial kinetic energy of the jacket, (2) the radial compressive potential energy generated by the core, and (3) the chemical energy released by the reactive inner core. They established a theoretical model for predicting the radial scattering velocity of jacket fragments and employed the powder burn model to simulate the deflagration reaction of the reactive core. Simulation results validated the accuracy of the theoretical analysis, demonstrating that this model can effectively and rapidly evaluate the fragmentation effect of RIC-PELEs. Numerical simulations and experimental investigations conducted by Meng et al. [29] revealed that an excessively small aspect ratio reduces the quantity of post-target fragments, consequently diminishing the damage ability of RIC-PELEs. Li et al. [30] found that, when the thickness of the steel target is matched appropriately, the reaction ratio of the reactive core increases significantly and the PELE produces combined kinetic and chemical energy destruction of the multi-layer interval aluminium witness targets.

Through experimental study and two-stage numerical simulations employing the ignition and growth model, Zhang et al. [31,32] elucidated the behind-target lateral enhancement mechanism of RIC-PELEs, demonstrating that the deflagration-induced energy release from the reactive core improves the maximum radial velocity of the jacket fragments by over 40%. RIC-PELEs also produce effective impact-induced deflagration damage against reinforced concrete targets. The perforation diameter in concrete targets exhibits a non-monotonic trend (an initial increase followed by a decrease) as the impact velocity and jacket thickness increase, but impact velocity is the dominant factor affecting collapse sizes [33]. By considering both radial rarefaction waves and the shock-induced energy release of the reactive core simultaneously, Zhang et al. [34,35] developed an aperture analytical model for RIC-PELE penetration into reinforced concrete. This model could effectively predict both the jacket deformation patterns and maximum perforation diameter in concrete targets. It was found that the deflagration reaction of the reactive core resulted in an increased degree of jacket deformation, which improved the maximum opening of the reinforced concrete target by 24.3%.

The fragmentation effect of a traditional PELE penetrating metal targets satisfies a strict geometric similarity law, as confirmed by Xu et al. [15]. However, there are no public reports of RIC-PELE similarity research. Analyzing the current related studies, it is easily observed that research on the lateral enhancement mechanism and damage effect of RIC-PELEs is gradually increasing. In these studies, it has been shown that the reactive core’s impact energy release effect has a crucial influence on jacket fragmentation. To reduce the blindness in RIC-PELE design and the expense of testing, it needs to be determined in detail whether the results of existing research can be scaled using the similarity law approach. Therefore, it is extremely important and meaningful to conduct a study on the similarity of the fragmentation effect of RIC-PELEs penetrating target plates.

In this study, the broken length of the jacket and the dispersion radius of jacket fragments behind the steel target were studied to determine the fragmentation effect of RIC-PELEs, and the similarity of that effect in penetrating a steel plate was analyzed based on dimensional theory. Subsequently, a simulation model of the RIC-PELE penetration and deflagration damage targets was established, and the reliability of the material parameters and simulation algorithms was verified using experimental results from the literature. Finally, jacket fragmentation and the distribution of jacket fragments behind the steel target were assessed using different scale models under certain conditions, and all simulation results were analyzed and discussed to explore the similarity of the fragmentation effect of RIC-PELEs penetrating a steel plate, verifying the theoretical analysis.

## 2. Theoretical Analysis of Similarity

Similar to the most common research methods, in conducting the similarity analysis, it is assumed that the scaled model and the prototype have the same material properties, the penetration process is very short and the heat conduction effect is ignored, and the influence of the material failure process is also neglected. Additionally, the scale effect caused by the strain rate effect of the material is not taken into account. This is because the strain rate sensitivity of the RIC-PELE jacket material and main target material in our research is relatively low. For the PTFE/Al, its strength is relatively small compared to the pressure generated by the collision between the projectile and the target. Therefore, in different scaled models, the change in strength caused by the variation in the strain rate is also very small. That is to say, its scale effect can be ignored.

### 2.1. Reaction Length of Reactive Inner Core

**Figure 1:**
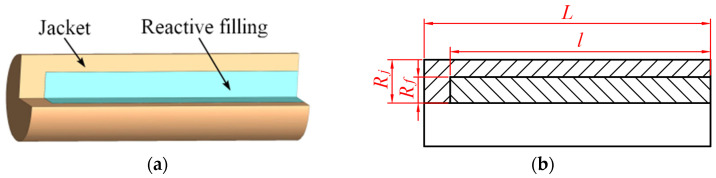
RIC-PELE schematic diagram: (**a**) 3D photograph; (**b**) dimensions.

Figure 1 presents a structural schematic diagram of a RIC-PELE; the jacket material is steel, and the reactive filling material is PTFE/Al. At the moment of the RIC-PELE’s vertical impact on the steel plate, backward-propagating shock waves are generated in the RIC-PELE. If the intensity of the shock wave in the inner core exceeds the minimum pressure *P_c_* that causes PTFE/Al to react, the PTFE/Al will deflagrate and release energy. When the shock wave propagates along the penetrator, its intensity gradually decays; therefore, the section of the inner core in which the shock wave intensity is higher than the threshold pressure *P_c_* is regarded as having undergone a complete deflagration reaction, while the remainder does not undergo any reaction, just like the inert material [28].

After the RIC-PELE penetrates the steel plate, the reaction length of the inner core *l_r_* can be expressed as a function of the geometric parameters of the penetrator and target plate, the material properties, and the threshold pressure *P_c_*:

(1) $$l_{r}=f(L,l,R_{j},R_{f},R_{t},H,\rho_{j},C_{j},S_{j},\rho_{f},C_{f},S_{f},\rho_{t},C_{t},S_{t},P_{c},v)$$

where *L* and *l* are the lengths of the jacket and core, respectively; *H* is the thickness of the circular steel plate; *v* is the impact velocity of the RIC-PELE; *R* presents the radius; *ρ*, *C*, and *S* present the density, sound velocity, and slope of the Rankine–Hugoniont line, respectively; and the subscripts *j*, *f*, and *t* denote the jacket, core, and steel plate, respectively (*R_j_* is the outer radius of the jacket).

Equation (1) contains 18 variables, of which there are 17 independent variables and 1 dependent variable. Among the 17 independent variables, *S_j_*, *S_f_*, and *S_t_* are dimensionless quantities. Taking *l*, *ρ_f_*, and *v* as independent dimensional fundamental quantities, according to Π theorem, Equation (1) can be written in dimensionless functional form:

(2) $$\frac{l_{r}}{l}=f(\frac{L}{l},\frac{R_{j}}{l},\frac{R_{f}}{l},\frac{R_{t}}{l},\frac{H}{l},\frac{\rho_{j}}{\rho_{f}},\frac{C_{j}}{v},\frac{C_{f}}{v},\frac{\rho_{t}}{\rho_{f}},\frac{C_{t}}{v},\frac{P_{c}}{\rho_{f}v^{2}},S_{j},S_{f},S_{t})$$

Under the condition that the RIC-PELE and target plate materials in the scaled model remain consistent with those in the prototype test, the physical quantities *ρ_j_*, *ρ_f_*, *C_j_*, *C_f_*, *ρ_t_*, *C_t_*, *S_j_*, *S_f_*, and *S_t_* in Equation (2) are specific constants. In addition, the threshold pressure *P_c_* required to activate PTFE/Al for a deflagration reaction is a constant value. Therefore, when the impact velocity remains unchanged, the dimensionless independent variables after the fifth one on the right side of Equation (2) remain constant. The expression for the dimensionless reaction length of the inner core can be further abbreviated as follows:

(3) $$\frac{l_{r}}{l}=f(\frac{L}{l},\frac{R_{j}}{l},\frac{R_{f}}{l},\frac{R_{t}}{l},\frac{H}{l})$$

In Equation (3), the dimensionless independent variables are the geometric parameters of both the RIC-PELE and the target plate. The core length ratio of the scaled model to the prototype is denoted as *γ*:

(4) $$\gamma =\frac{{\left(l\right)}_{m}}{{\left(l\right)}_{p}}$$

where the subscripts *m* and *p* denote the scaled model and the prototype, respectively. To ensure that (*l_r_*/*L*)*_m_* = (*l_r_*/*L*)*_p_* holds, the following must be satisfied:

(5) $$\left\{\begin{array}{l} {\left(\frac{L}{l}\right)}_{m}={\left(\frac{L}{l}\right)}_{p}\\ {\left(\frac{R_{j}}{l}\right)}_{m}={\left(\frac{R_{j}}{l}\right)}_{p}\\ {\left(\frac{R_{f}}{l}\right)}_{m}={\left(\frac{R_{f}}{l}\right)}_{p}\\ {\left(\frac{R_{t}}{l}\right)}_{m}={\left(\frac{R_{t}}{l}\right)}_{p}\\ {\left(\frac{H}{l}\right)}_{m}={\left(\frac{H}{l}\right)}_{p} \end{array}\right.$$

That is, as long as Equation (5) holds, when the RIC-PELE penetrates the steel plate, the reaction length of its inner core complies with the geometric similarity law.

### 2.2. Broken Length of Jacket

According to the research of Xu et al. [15], when analyzing the similarity of the fragmentation effect of conventional PELE jackets, in addition to the geometric parameters of the penetrator and target plate, the influences of the density, elasticity modulus, Poisson’s ratio, the strength of the penetrator and target plate materials, and the strain of the penetrator jacket material on the jacket fragmentation need to be considered. Therefore, when analyzing the similarity problem of the RIC-PELE jacket fragmentation effect, the above physical quantities still need to be taken into account. Additionally, the reaction length of the inner core and the large amount of chemical energy released during the reaction are also included. In our dimensional analysis, the influence of the reaction length of the inner core on the jacket fragmentation is reflected through the mass of the reactive material that undergoes reaction. The mechanism of chemical energy acting on the jacket can be analyzed by referring to the fragment scattering mechanism of a cylindrical fragment warhead [28]. The mass of reactive material undergoing a full reaction is denoted as *m*, and the energy released from the unit mass of reactive material is denoted as *Q*. The function of the broken length of the jacket *L_b_* after perforating the steel plate is expressed as follows:

(6) $$L_{b}=f(L,l,R_{j},R_{f},R_{t},H,\rho_{j},E_{j},\mu_{j},\sigma_{j},\varepsilon_{j},\rho_{f},E_{f},\mu_{f},\sigma_{f},\rho_{t},E_{t},\mu_{t},\sigma_{t},v,m,Q)$$

where *E*, *μ*, *σ*, and *ε* are the elasticity modulus, Poisson’s ratio, strength, and strain of the material, respectively.

In Equation (6), *μ_j_*, *ε_j_*, *μ_f_*, and *μ_t_* are dimensionless quantities, while *l*, *ρ_f_*, and *v* are still taken as independent dimensional fundamental quantities, according to the Π theorem. Equation (6) can be written in dimensionless functional form:

(7) $$\frac{L_{b}}{l}=f(\frac{L}{l},\frac{R_{j}}{l},\frac{R_{f}}{l},\frac{R_{t}}{l},\frac{H}{l},\frac{\rho_{j}}{\rho_{f}},\frac{E_{j}}{\rho_{f}v^{2}},\frac{\sigma_{j}}{\rho_{f}v^{2}},\frac{E_{f}}{\rho_{f}v^{2}},\frac{\sigma_{f}}{\rho_{f}v^{2}},\frac{\rho_{t}}{\rho_{f}},\frac{E_{t}}{\rho_{f}v^{2}},\frac{\sigma_{t}}{\rho_{f}v^{2}},\frac{m}{\rho_{f}l^{3}},\frac{Q}{v^{2}},\mu_{j},\varepsilon_{j},\mu_{f},\mu_{t})$$

By the same token, when the RIC-PELE and target plate materials in each scale model are exactly the same, the values of physical quantities such as *ρ_j_*, *E_j_*, *σ_j_*, *μ_j_*, *ε_j_*, *ρ_f_*, *E_f_*, *σ_f_*, *μ_f_*, *ρ_t_*, *E_t_*, *σ_t_*, *μ_t_*, and *Q* in Equation (7) remain unchanged. Under identical impact velocity, Equation (7) can be simplified as follows:

(8) $$\frac{L_{b}}{l}=f(\frac{L}{l},\frac{R_{j}}{l},\frac{R_{f}}{l},\frac{R_{t}}{l},\frac{H}{l},\frac{m}{\rho_{f}l^{3}})$$

The *m* in Equation (8) can be expressed as follows:

(9) $$m=\rho_{f}\pi R_{f}^{2}l_{r}$$

Substituting Equation (9) into Equation (8) and simplifying, the following can be obtained:

(10) $$\frac{L_{b}}{l}=f(\frac{L}{l},\frac{R_{j}}{l},\frac{R_{f}}{l},\frac{R_{t}}{l},\frac{H}{l},\frac{\pi R_{f}^{2}l_{r}}{l^{3}})$$

In Equation (10), *π* is a dimensionless quantity, and its value remains unchanged in each scale model. Therefore, the above equation can be further simplified as follows:

(11) $$\frac{L_{b}}{l}=f(\frac{L}{l},\frac{R_{j}}{l},\frac{R_{f}}{l},\frac{R_{t}}{l},\frac{H}{l},\frac{R_{f}^{2}l_{r}}{l^{3}})$$

In Equation (11), the physical significance of the last dimensionless independent variable is not clear. Combined with the properties of dimensional analysis, the following transformation can be carried out:

(12) $$\frac{R_{f}^{2}l_{r}}{l^{3}}\to \frac{R_{f}^{2}l_{r}}{l^{3}}/\frac{R_{f}^{2}}{l^{2}}=\frac{l_{r}}{l}$$

Then, Equation (11) can be further written as follows:

(13) $$\frac{L_{b}}{l}=f(\frac{L}{l},\frac{R_{j}}{l},\frac{R_{f}}{l},\frac{R_{t}}{l},\frac{H}{l},\frac{l_{r}}{l})$$

As can be seen from the analysis in Section 2.1, if Equation (5) holds, then (*l_r_*/*L*)*_m_* = (*l_r_*/*L*)*_p_* must also hold. Thus, the following equation can be obtained:

(14) $${\left(\frac{L_{b}}{l}\right)}_{m}={\left(\frac{L_{b}}{l}\right)}_{p}$$

It is evident that the broken length of the RIC-PELE jacket after perforating the steel plate satisfies the geometric similarity law.

### 2.3. Dispersion Radius of Jacket Fragments Behind the Steel Plate

The distance between the rear-effect aluminum plate and the circular steel plate is denoted as *D*, and the dispersion radius of the jacket fragments behind the front target is denoted as *R_i_*. *R_j_*, *ρ_f_*, and *v* were selected as the independent dimensional fundamental quantities. Similarly, under the condition that the RIC-PELE and target plate materials are identical and the impact velocity is fixed, the dimensionless dispersion radius of the jacket fragments behind the target can be deduced as follows:

(15) $$\frac{R_{i}}{R_{j}}=f(\frac{L}{R_{j}},\frac{l}{R_{j}},\frac{R_{f}}{R_{j}},\frac{R_{t}}{R_{j}},\frac{H}{R_{j}},\frac{D}{R_{j}},\frac{m}{\rho_{f}R_{j}^{3}})$$

By substituting Equation (9) into Equation (15) and simplifying, the following is obtained:

(16) $$\frac{R_{i}}{R_{j}}=f(\frac{L}{R_{j}},\frac{l}{R_{j}},\frac{R_{f}}{R_{j}},\frac{R_{t}}{R_{j}},\frac{H}{R_{j}},\frac{D}{R_{j}},\frac{R_{f}^{2}l_{r}}{R_{j}^{3}})$$

In accordance with the customary representation in the study of penetration problems, Equation (16) can be written as follows:

(17) $$\frac{R_{i}}{d_{j}}=f(\frac{L}{d_{j}},\frac{l}{d_{j}},\frac{R_{f}}{d_{j}},\frac{R_{t}}{d_{j}},\frac{H}{d_{j}},\frac{D}{d_{j}},\frac{R_{f}^{2}l_{r}}{d_{j}^{3}})$$

where *d_j_* is the outer diameter of the RIC-PELE jacket. The last dimensionless independent variable in Equation (17) can be transformed as follows:

(18) $$\frac{R_{f}^{2}l_{r}}{d_{j}^{3}}\to \frac{R_{f}^{2}l_{r}}{d_{j}^{3}}/\frac{R_{f}^{2}}{d_{j}^{2}}=\frac{l_{r}}{d_{j}}$$

Then, Equation (17) can be further written as follows:

(19) $$\frac{R_{i}}{d_{j}}=f(\frac{L}{d_{j}},\frac{l}{d_{j}},\frac{R_{f}}{d_{j}},\frac{R_{t}}{d_{j}},\frac{H}{d_{j}},\frac{D}{d_{j}},\frac{l_{r}}{d_{j}})$$

According to Equation (19), the ratio of the dimensionless dispersion radius of the jacket fragments behind the target in the scaled model to that in the prototype can be given as follows:

(20) $$\frac{{\left(\frac{R_{i}}{d_{j}}\right)}_{m}}{{\left(\frac{R_{i}}{d_{j}}\right)}_{p}}=\frac{{\left[f(\frac{L}{d_{j}},\frac{l}{d_{j}},\frac{R_{f}}{d_{j}},\frac{R_{t}}{d_{j}},\frac{H}{d_{j}},\frac{D}{d_{j}},\frac{l_{r}}{d_{j}})\right]}_{m}}{{\left[f(\frac{L}{d_{j}},\frac{l}{d_{j}},\frac{R_{f}}{d_{j}},\frac{R_{t}}{d_{j}},\frac{H}{d_{j}},\frac{D}{d_{j}},\frac{l_{r}}{d_{j}})\right]}_{p}}$$

The scaled model and the prototype satisfy the geometric similarity condition, and Equation (20) can be simplified as follows:

(21) $$\frac{{\left(\frac{R_{i}}{d_{j}}\right)}_{m}}{{\left(\frac{R_{i}}{d_{j}}\right)}_{p}}=\frac{{\left[f(\frac{l_{r}}{d_{j}})\right]}_{m}}{{\left[f(\frac{l_{r}}{d_{j}})\right]}_{p}}$$

From the analysis in Section 2.1, we know that the reaction length of the inner core satisfies the geometric similarity law; thus, the following can be obtained:

(22) $${\left(\frac{l_{r}}{d_{j}}\right)}_{m}={\left(\frac{l_{r}}{d_{j}}\right)}_{p}$$

Therefore, for the dimensionless dispersion radius of the jacket fragments behind the target, it should be

(23) $${\left(\frac{R_{i}}{d_{j}}\right)}_{m}={\left(\frac{R_{i}}{d_{j}}\right)}_{p}$$

Obviously, when the RIC-PELE penetrates the steel plate, the dispersion radius of the jacket fragments behind the target satisfies the geometric similarity law.

Through theoretical analysis, it was found that the independent variables in the dimensionless function expressions of the two physical quantities used for evaluating the fragmentation effect of RIC-PELEs are only geometric dimensionless quantities. This indicates that the fragmentation effect of the RIC-PELE penetrating the steel plate satisfies the geometric similarity law.

## 3. Numerical Simulation

### 3.1. Numerical Simulation Algorithm

The smooth particle hydrodynamics (SPH) algorithm is a meshless particle method [36] that has been widely used to simulate problems such as hypervelocity impact [37,38], hypervelocity penetration [39], explosion damage [40], and the formation and distribution of fragments [41,42,43]. In the SPH method, the state of the system is represented by arbitrarily distributed particles that contain all of the computational information, such as mass, velocity, and elasticity modulus. These particles are free to move according to interactions between them or with external forces. The SPH method is suitable for simulating large deformation problems such as fragmentation, spallation of solids, crack extension, or brittle fracture; it has the advantage of overcoming the defect of the traditional Lagrange finite element mesh (FEM) method, where deleting part of the mesh can lead to distorted results or termination of computation due to excessive model distortion. When using this method, the setting of the initial particle spacing, i.e., the particle size, has a significant influence on the simulation results. If the value is too large, it will reduce the accuracy of the results and fail to display detailed features and local information; if it is too small, sufficient forces cannot be provided to the particles within the search domain, thereby reducing the calculation accuracy [37]. Therefore, a suitable particle size needs to be selected. However, for large models, the number of particles that may be required to ensure computational accuracy is huge, and the procedure takes a long time to run and requires high computational performance.

**Figure 2:**
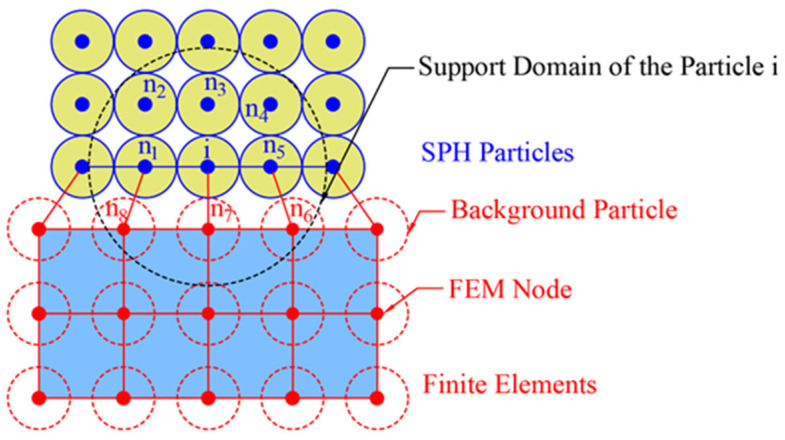
Principle of the SPH–FEM coupling method.

Compared with the SPH algorithm, the FEM algorithm has a shorter running time and could significantly improve the computational efficiency. Therefore, in order to make full use of the advantages of the SPH and FEM algorithms, they could be coupled to simulate the damage and dynamic response of structures or materials under impact loads [44,45]. The schematic diagram of the coupling principle between SPH particles and FEM is shown in Figure 2. The smaller blue solid circles represent SPH particles, while the larger dashed circle around particle i and smaller red dashed circles represent the support domains of the SPH particle i and the background particles set at the FEM nodes, respectively. The background particles have the properties of SPH particles, and their variables are consistent with those of the corresponding FEM nodes, such as the mass, stress, velocity, and position of the particles. That is, the FEM nodes can be regarded as SPH particles in the background particle mode [36,46].

When conducting the numerical calculation, in each time step, the physical quantities of the finite element node are transferred to the corresponding background particle. The SPH particle located at the interface, its velocity and position will be transmitted to the corresponding finite element node after completing each time step. When integrating the velocity, displacement and energy of particle i, SPH particles n_1_, n_2_, n_3_, n_4_ and n_5_, as well as finite element nodes n_6_, n_7_ and n_8_, must be taken into account simultaneously. The background particles are searched via the SPH particles, but during this process, they do not perform the SPH numerical integration themselves; the data update is accomplished by the corresponding finite element nodes [36].

For the SPH part and the FEM part, the contact between them is defined through the node to face method, which transmits force and velocity. The part defined with SPH particles is the slave part, and the master part defined with finite elements. The velocity of the SPH particles in contact with the FEM part is denoted as *u*’, and the velocity of the finite elements in contact with the SPH part is denoted as *u*. The contact velocity between the SPH and FEM parts satisfies *u*’ = *u*.

When the materials of the slave and main part undergo deformation and failure under the action of force, the SPH particles of the slave part will not be deleted, but the finite element meshes of the main part will be deleted when the deformation exceeds the set value.

To verify the theoretical analysis results of the similarity law, the processes of projectiles’ penetration into steel plates were simulated using ANSYS/Autodyn software. The SPH–FEM coupling algorithm was adopted, i.e., the SPH algorithm was used for the inner core and jacket, and the Lagrange algorithm for the metal targets. This could better simulate the fragmentation process of the penetrator and the distribution of the jacket fragments. In the modeling, the PTFE/Al core adopted a segmented method. The reaction length of the PTFE/Al core was determined based on the intensity of the shock wave propagating within it when hitting the target—that is, the core with shock wave pressure less than the critical activation threshold of the reactive material (3.6 GPa) was regarded as inert (unactivated reaction), while the core with shock wave pressure greater than this value was regarded as reactive (activated reaction) [47]. Consequently, it was necessary to treat PTFE/Al as an inert material and analyze the intensity of the shock wave in the core via the Lagrange algorithm before simulating the RIC-PELE’s penetration into the steel plate with the coupled SPH–FEM algorithm. When analyzing the propagation of the shock wave, the Lagrange algorithm was used instead of the SPH–FEM coupling algorithm, because the SPH algorithm itself has some deficiencies. When a shock wave passes through the interface of two media with obvious density differences, physical quantities such as density and pressure may exhibit abnormal phenomena. It is not guaranteed that the pressure variation in the shock wave in the PTFE/Al inner core can be obtained precisely.

### 3.2. Material Models and Parameters

The materials of the RIC-PELE jacket, the front target plate, and the witness target were 30CrMnSiNi2A steel, RHA steel, and 2024 aluminum, respectively. In the numerical simulations, a shock equation of state (EOS) and the Johnson–Cook strength model were used for 30CrMnSiNi2A steel, 2024 aluminum, and unactivated PTFE/Al. For the activated PTFE/Al with the deflagration reaction, the powder burn model was used for the EOS, while the strength model was consistent with that of the unactivated part. Shock EOS and the von Mises strength model were used for RHA steel. The failure behavior of materials is closely related to the loading conditions to which they are subjected. In order to accurately describe the whole process of a RIC-PELE acting on double-spaced metal targets, the principal stress failure model was chosen for all materials used in this paper. Specifically, material failure will only occur when either the maximum tensile principal stress or shear stress exceeds the limit value of the material itself [48]. For the Lagrange algorithm, the erosion must be set for the corresponding material. In this paper, the erosion type “Geometric Strain” was selected for all materials.

All material parameters for the jacket and witness target were determined as described by Li et al. [30], Yu et al. [49], Xin et al. [50], Chu et al. [51], and Yan et al. [52]. The material parameters for the inner core were obtained from Liang [53], Ren et al. [38], and Li et al. [30]. All parameters are listed in Table 1, Table 2 and Table 3. The material parameters for the front target were taken from the Autodyn material library, and the specific values are no longer listed here.

### 3.3. Numerical Simulation Model for Similarity Analysis

**Figure 3:**
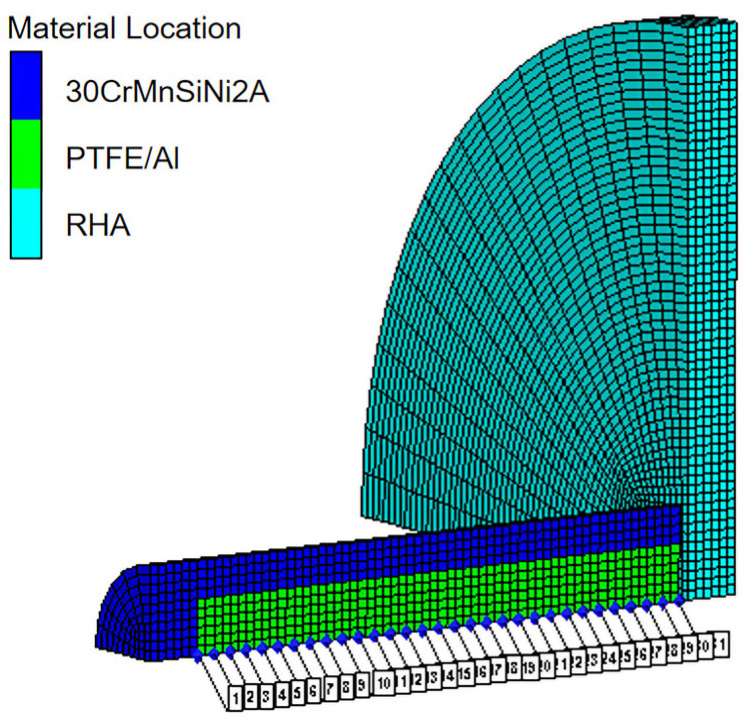
Lagrange finite element simulation model.

Due to the central symmetry of the geometric model of a RIC-PELE vertically penetrating a steel plate, a 1/4 simplified model was adopted in the simulation to save calculation time. When using the Lagrange algorithm to analyze the intensity of the shock wave generated by collision in the core, the aluminum witness plate was not required. The finite element model is shown in Figure 3, where the number of meshes in the axial and radial directions of the inner core was 60 and 6, respectively. At the contact surface between the inner core and the jacket, there were six circumferential meshes. The mesh size of the jacket in the axial and radial directions was the same as that of the inner core, and the circumferential mesh number was six. The jacket and inner core were connected by “join” to achieve a common node. For the steel plate, the mesh of the projectile impact and nearby area was denser; in the 1/4 model, the size of this area was 2*R_j_*. If the axial and radial mesh lengths of the inner core were denoted as *e*, then the minimum mesh size of this densification area was *e*. For different scaled models, the mesh sizes were different but the number of meshes was the same, i.e., the mesh had the same scaling ratio as the model. To record the variation in shock wave pressure at different positions in the core over time, 31 observation points were set at equal intervals on the central axis of the inner core. The 1st and 31st observation points were located on the bottom and top end-faces of the inner core, respectively, as shown in Figure 3.

**Figure 4:**
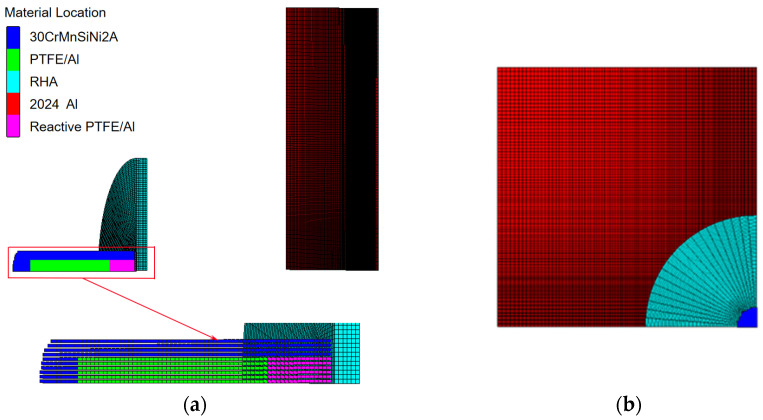
SPH–FEM coupled simulation model: (**a**) oblique view; (**b**) front view.

The SPH–FEM coupling algorithm was utilized to analyze the fragmentation effect when a RIC-PELE vertically penetrated a steel plate, and an aluminum witness plate was added, as shown in Figure 4. The meshing of the steel plate was exactly the same as that in Figure 3, and the minimum mesh size in the refined area was also *e*. The SPH particle size of the PELE jacket and inner core was also *e*. To enhance the computational efficiency, the aluminum witness plate was divided by a gradient mesh, with the central area refined. The side length of the refined area was 75 mm, and the mesh size was 1.5 mm. In the 1/4 model, it was only necessary to ensure that the side length of the aluminum witness plate was greater than the scattering radius of the jacket fragments of the penetrator, so its geometric dimensions were not strictly required and did not need to be changed proportionally during simulation. The thickness of the aluminium witness plate was 1.5 mm in all of the simulations of the different scaled models, because its thickness did not affect the scattering radius of the jacket fragments behind the front target.

The dimensions of the model for conducting convergence verification were as follows: the length of the jacket was 33 mm, and the outer radius was 5 mm; the length of the reactive core was 30 mm, and the radius was 3 mm; the radius of the steel target was 30 mm and its thickness was 3 mm (which was the reference benchmark model used in this study). At the impact speed of 1500 m/s, When *e* = 0.4, 0.5, and 0.6 mm, the dispersion radius of the jacket fragments on the aluminum witness target was 39 mm, 40 mm, and 39.98 mm respectively. The dispersion radius of the jacket fragments was not significantly different, indicating that the calculation results were convergent. However, when *e* = 0.4 mm, the calculation time significantly increased, being several times that of when *e* = 0.5 mm. Taking all factors into consideration, choosing *e* = 0.5 mm was the most appropriate option.

### 3.4. Validation of Numerical Simulation

Li et al. [30] conducted ballistic impact experiments to determine the perforation size produced by the RIC-PELE on the main target plate and the damage size on the witness target. In our research, the materials of the projectile and targets were exactly the same as those in this literature, i.e., the jacket, inner core, main target, and witness target materials were 30CrMnSiNi2A steel, PTFE/Al, RHA steel, and 2024 aluminum, respectively. To verify the reliability of the model and material parameters used in the simulations, one set of experimental results from the literature [30] (the experiment number is 2–1 in this paper) was selected for comparative analysis. The caliber of the RIC-PELE was 30 mm, its length was 100 mm, and the inner diameter of its jacket was 18 mm. The length and width of the RHA steel target were both 500 mm, and the thickness was 20 mm. The length and width of the 2024 aluminum target were both 1000 mm, and the thickness was 3 mm. The distance between the main target and the witness target was 200 mm. The RIC-PELE hit the main target at a speed of 970 m/s.

**Figure 5:**
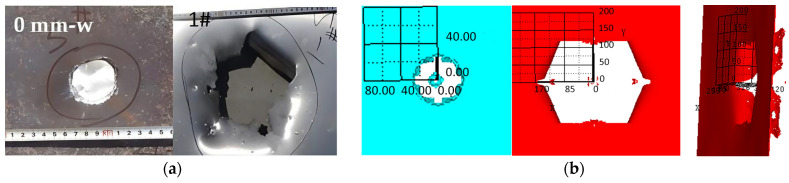
Comparison of main and witness target damage between the experiment and the numerical simulation: (**a**) experimental results [30]; (**b**) simulation results.

The simulation verification adopted a 1/4 model. When using the Lagrange algorithm to analyze the intensity of the collision shock wave, the projectile and the steel target grids were divided according to the method described in Section 3.3. The side length of the encryption area of the steel target was 30 mm, and the mesh size was 1.5 mm. At an impact speed of 970 m/s, the reaction length of the inner core was determined to be 28 mm through Lagrange simulation. Based on this, the SPH–FEM model was established to analyze the damage effect of the target plates. In this model, the SPH particle size of the projectile was 1.5 mm, and the grid division of the steel target was the same as before. For the aluminum target, a gradient grid was used as well, with the side length of the denser area being 75 mm and the mesh size being 1.5 mm. Figure 5 shows a comparison between the experimental and numerical simulation results. It is worth noting that, in the original literature, the steel target and aluminum target only captured the local areas that contained the damaged zones. To facilitate comparison, the simulation results were also presented in this manner. For a detailed comparison of damage indicators, please refer to the data and analysis presented below.

It can be seen from the experimental results in Figure 5a that the perforation shape of the RIC-PELE on the main target plate is approximately circular. After penetrating the main target plate, it caused severe central rupture and perforation damage to the aluminum witness plate, and a distinct bulge was formed on the back. This indicates that the reactive material core was significantly fragmented after perforating the main target plate, and a deflagration reaction was activated under the pressure that it received during the penetration process. Under the combined action of the squeezing and deflagration reaction of the core, the radial scattering velocity of the jacket fragments was increased, resulting in a large-scale damage zone on the witness target.

From the simulation results in Figure 5b, it can be seen that the perforation shape on the main target plate is a circle. After penetrating the steel plate, the RIC-PELE caused severe central rupture and perforation damage to the aluminum witness plate and formed an evident bulge on the back. The damage patterns of the primary target and the witness target were identical to the experimental results. In the simulation, the damage zone of the aluminium witness plate was more symmetrical, because the RIC-PELE perpendicularly penetrated the target plate, while the posture of the penetrator might have been tilted to a certain extent when it hit the steel plate in the experiment, as proven by the nearly circular perforation on the steel plate in Figure 5a. A detailed comparison of the simulation and experimental results is shown in Table 4. The perforation diameters on the main target plate were almost equal. For the aluminum witness plate, taking half of the maximum damage size as the dispersion radius *R_i_* and normalizing it by the penetrator diameter *d_j_*, the experimental and simulated values of *R_i_*/*d_j_* are 6.0 and 5.7, respectively. The absolute value of the numerical simulation error is 5%, which is within an acceptable error range of 10%.

The perforation size on the main target, the damage model of the two targets, and the dimensionless damage radius *R_i_*/*d_j_* (dimensionless dispersion radius of the jacket fragments) were compared. It is not difficult to see that the simulation results are highly consistent with the experimental ones, indicating that the algorithm model and material parameters used in this paper could effectively simulate the fragmentation of the jacket and the scattering of the jacket fragments.

## 4. Results and Discussion

### 4.1. Numerical Simulation Scheme for Similarity Analysis

The above simulation model was applied to study the geometric similarity law of the fragmentation effect of the RIC-PELE penetrating a steel plate. The model with a magnification ratio *γ* = 1 was used as the reference benchmark, and the dimensions of the model were as follows: the jacket length and outer radius were 33 mm and 5 mm, the inner core length and radius were 30 mm and 3 mm, and the radius and thickness of the steel plate were 30 mm and 3 mm, respectively, and the distance between the steel plate and the aluminium witness plate was 65 mm. In addition, simulation models with magnification ratios of *γ* = 2, *γ* = 3, *γ* = 4, and *γ* = 5 were established. The geometric parameters and mesh sizes of the simulation models with different scales are shown in Table 5.

### 4.2. Normalization of the Reaction Length of the Reactive Inner Core

**Figure 6:**
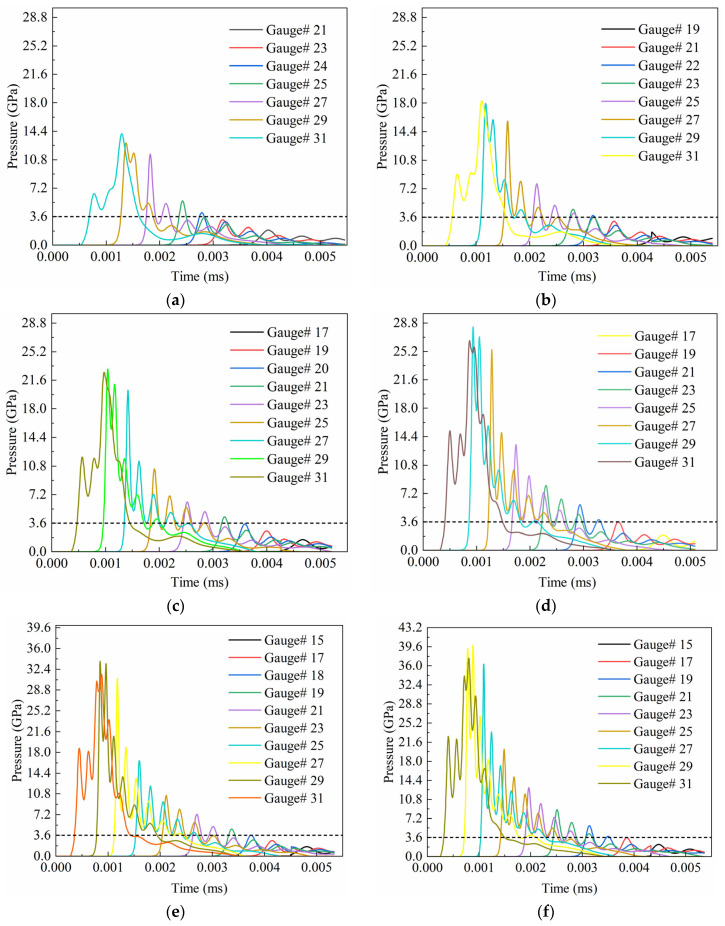
Pressure–time history of the observation points on the central axis of the reactive inner core with *γ* = 1: (**a**) 900 m/s; (**b**) 1100 m/s; (**c**) 1300 m/s; (**d**) 1500 m/s; (**e**) 1700 m/s; (**f**) 1900 m/s.

When the RIC-PELE with a magnification ratio of *γ* = 1 penetrated the steel plate at different speeds, the pressure–time history curves of some selected observation points on the central axis of the inner core were as shown in Figure 6. In the simulation model, observation point 31 was located at the most anterior part of the center axis of the inner core, i.e., at the interface where it impacted with the steel plate. The peak pressure of the collision shock wave at this location was the largest for the six velocity conditions, and the peak pressure of the shock wave on the center axis decreased as the shock wave propagated to the rear of the core. The distance between neighbouring observation points in the model with magnification ratio *γ* = 1 was 1 mm. As demonstrated in Figure 6a, the peak pressure at observation point 24 was slightly higher than 3.6 GPa, while that at observation point 23 was slightly lower than 3.6 GPa. Therefore, it is approximated that the peak pressure of the shock wave at a distance of 7.5 mm from the front end of the inner core is 3.6 GPa, i.e., the length of the PTFE/Al material for the deflagration reaction is 7.5 mm. As shown in Figure 6b–f, when the impact velocities were 1100, 1300, 1500, 1700, and 1900 m/s, the observation points with the peak shock wave pressure of 3.6 GPa were 22, 20, 19, 18, and 17, respectively; that is, the lengths of the PTFE/Al material undergoing the deflagration reaction at the corresponding impact velocity were 9, 11, 12, 13, and 14 mm, respectively. The inner core deflagration reaction lengths of other scaled RIC-PELEs can also be obtained via this method.

**Figure 7:**
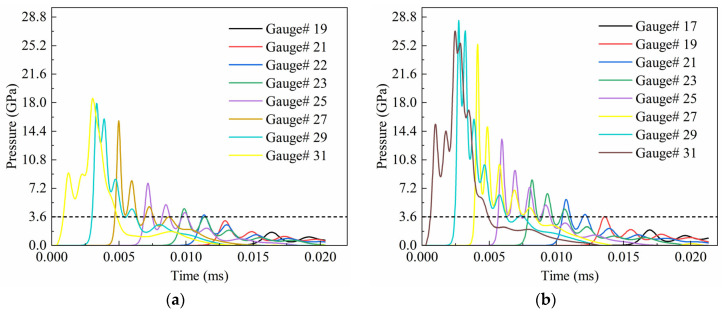
Pressure–time history of the observation points on the central axis of the reactive inner core with *γ* = 4: (**a**) 1100 m/s; (**b**) 1500 m/s.

When the RIC-PELE with *γ* = 4 penetrated the steel plate, the pressure–time history curves of the observation points on the central axis of the inner core were as presented in Figure 7. The observation points selected in Figure 7a are exactly the same as those in Figure 6b, while those selected in Figure 7b are exactly the same as those in Figure 6d. When the impact velocities are the same, it is easy to find that the pressure–time history curves of the impact shock waves at the corresponding observation points on the inner core center axes of the different scaled penetrators are essentially identical, and the observation points with a peak pressure of 3.6 GPa are also identical. Only the corresponding times on the horizontal coordinate are significantly different, but this has no significance in this study. In fact, the time corresponding to the peak pressure at the same observation point also varies strictly according to the model scale, because the propagation of shock waves in geometrically similar solid media has timescale similarity, i.e., the dimensionless times are equivalent; this conclusion has been confirmed by Zhao [54]. For example, the time corresponding to the peak pressure at each observation point in Figure 7a is approximately four times that of Figure 6b, and the error exists because of the different initial gap between the penetrator and the target plate during the simulation. Since the distance between neighbouring observation points varies strictly in accordance with the magnification ratio of the model, for the penetrator with *γ* = 4, the deflagration reaction length of its inner core is four times that of the reference benchmark.

The variation laws of other scaled-up models under all speed conditions are the same as those of the aforementioned *γ* = 4. It is indicated that the deflagration reaction length of the inner cores follows a strictly proportional variation when geometrically similar RIC-PELEs penetrate the steel plate at an identical impact velocity, which corresponds to the magnification ratio of the geometric dimensions of the model.

**Figure 8:**
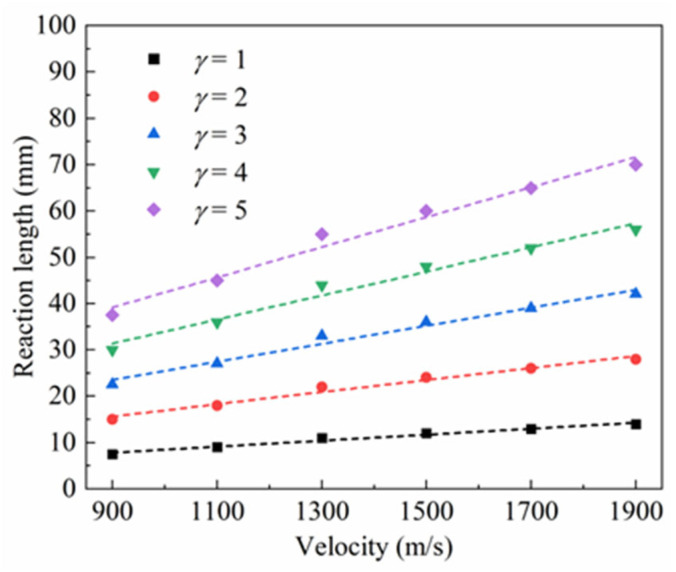
The variation in the reaction length of the inner core with impact velocity.

Figure 8 presents the variation laws of the deflagration reaction length of the inner core with the impact speed. It can be seen that within the impact speed range of 900 to 1900 m/s, the deflagration reaction lengths of the inner cores of the five scaled RIC-PELEs increase approximately linearly with the increase in velocity. The reason for this is that, within the studied velocity range, the higher the velocity, the greater the pressure at the projectile–target collision interface, which leads to the collision shock wave propagating backward along the inner core, and the distance required for its intensity to decay to the threshold pressure *P_c_* increases [55,56]. At the same impact speed, the greater the magnification ratio of the model, the longer the deflagration reaction length of the inner core.

**Figure 9:**
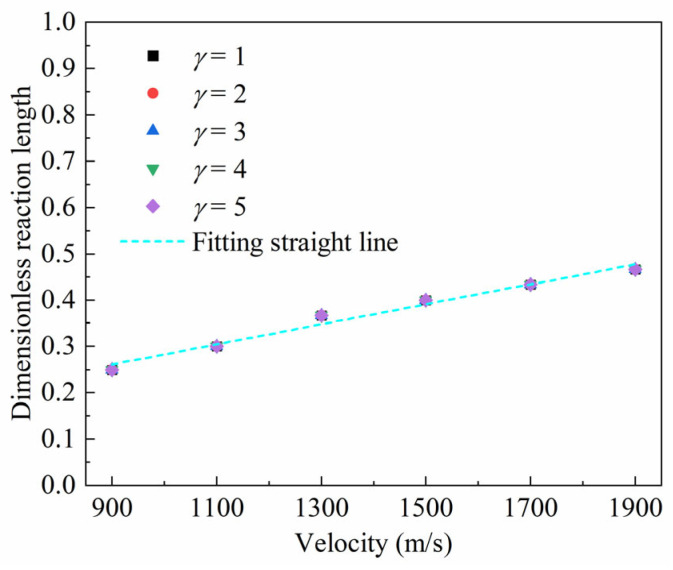
The variation in the dimensionless reaction length of the inner core with impact velocity.

The results in Figure 8 were normalized according to the left term of Equation (3) to obtain the variation law of the dimensionless reaction length of the inner core with the impact velocity, as shown in Figure 9. At the same impact velocity, the dimensionless deflagration reaction lengths of the inner cores of different scaled RIC-PELEs coincide at one point, which indicates that the reaction length of the reactive inner core satisfies a strict geometric similarity law when the RIC-PELE penetrates the steel plate. As the impact speed increases, the dimensionless deflagration reaction length of the inner core also increases linearly. To visually analyze the influence of velocity on the dimensionless dependent variable, in their relationship plot, the velocity on the horizontal coordinate carries a unit. Therefore, when expressing this relationship with a mathematical equation, a quantity *v*_1_ (*v*_1_ = 1 m/s) needs to be introduced to eliminate the velocity unit, ensuring that the right side of the equation is also dimensionless. Through juxtaposed fitting, a straight line describing their relationship is given in Equation (24):

(24) $$\frac{l_{r}}{l}=0.07+(2.17\times 10^{-4})\frac{v}{v_{1}}$$

where *v* represents the impact velocity of the RIC-PELE, and the correlation coefficient *R^2^* = 0.98. Equation (24) can be further written as follows:

(25) $$\frac{l_{r}}{l}=0.07+\frac{0.217v}{10^{3}v_{1}}$$

Referring to the method used by Anderson Jr. et al. [57] to represent the dimensionless penetration depth of long rod projectiles, the juxtaposed-fitting straight line describing the relationship between the dimensionless reaction length and the impact velocity is ultimately written as follows:

(26) $$\frac{l_{r}}{l}=0.07+0.217\frac{v}{v_{r}}$$

where *v_r_* is a reference velocity, and its value is 1000 m/s. In Equation (26), the fitting coefficient 0.07 represents the intercept. However, it has no actual physical meaning. It merely represents the intersection point of the extension line of the fitting straight line obtained within the impact velocity range of 900–1900 m/s and the vertical coordinate axis. Because when the dimensionless velocity *v*/*v_r_* = 0, the projectile is stationary and the reactive inner core cannot undergo any reaction, the dimensionless reaction length cannot be 0.07. The fitting coefficient 0.217 represents the slope, which reflects the linear enhancement effect of the impact velocity on the reaction length of the reactive inner core. The magnitude of this coefficient indicates the sensitivity of the dimensionless reaction length to the change in velocity.

### 4.3. Normalization of the Broken Length of the Jacket

**Figure 10:**
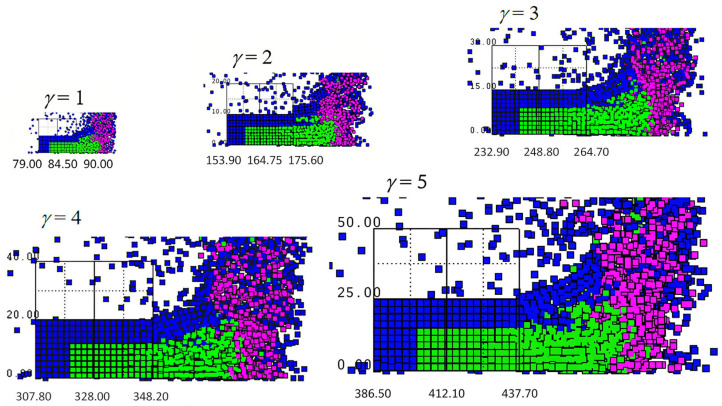
The jacket fragmentation of different scale models at the impact speed of 1500 m/s.

Figure 10 presents the jacket fragmentation behaviors of different scale models at a speed of 1500 m/s (1/4 simulation diagram). When *γ* = 1, 2, 3, 4, and 5, the remaining lengths of the jackets are 11, 21.7, 31.8, 40.4, and 51.2 mm, respectively, thereby revealing that the broken lengths of the jackets are 22, 44.3, 67.2, 91.6, and 113.8 mm, respectively. The variation ratio of broken length is approximately equal to the magnification ratio of the model. Under other impact speeds, the variation law of the broken length of the jacket with different scales is the same as that at 1500 m/s.

**Figure 11:**
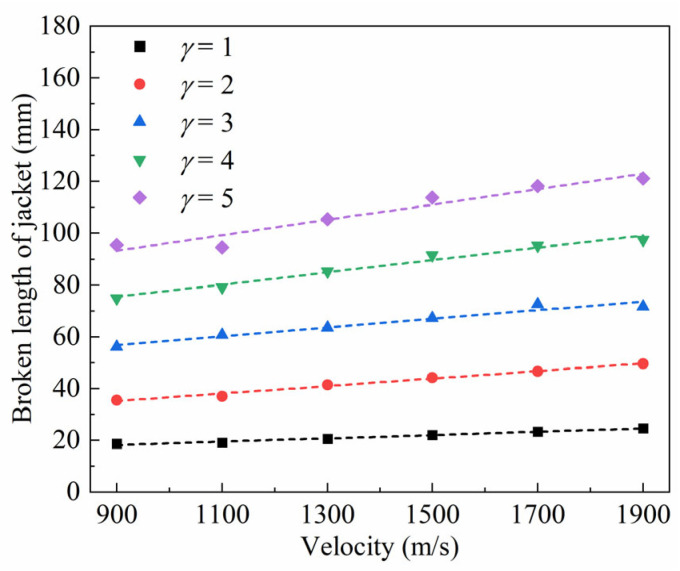
The variation in the broken length of the jacket with the impact velocity.

Figure 11 presents the variation laws of the broken length of the jacket with the impact speed when five magnification ratios of RIC-PELE penetrate the steel plate. Within the studied speed range, the broken length of jackets with the same magnification ratio increases linearly with the increase in impact speed. The pressure at the projectile–target collision interface increases with the impact velocity, resulting in an increase in the deflagration reaction rate and reaction ratio of the reactive inner core, and enhancing the chemical energy released by the reaction of the reactive material [49]. Simultaneously, the degree of radial expansion of the inner core material and the length of radial expansion increase with the rise in the impact speed [4]. Consequently, under the combined effects of greater chemical energy release and more extensive radial expansion of the core material, the broken length of the jacket increases. At the same impact velocity, the larger the scaling ratio of the model, the longer the broken length of the outer jacket.

**Figure 12:**
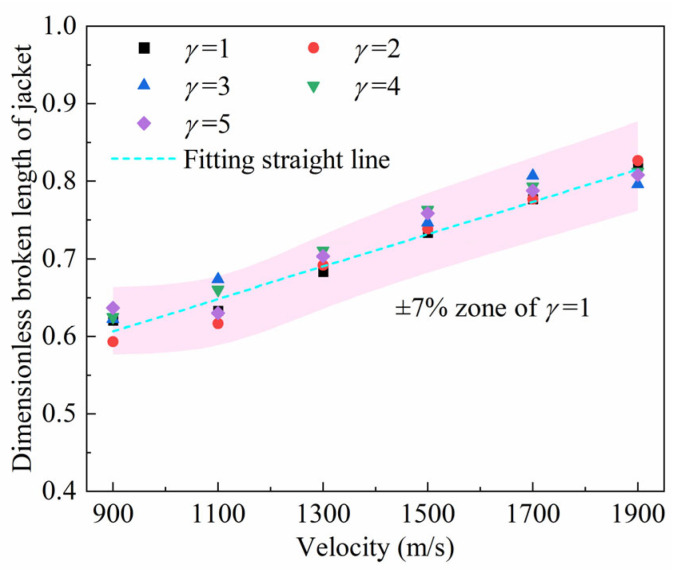
The variation in the dimensionless broken length of the jacket with the impact velocity.

The broken lengths of the jackets in Figure 11 were normalized according to the left term of Equation (13), as shown in Figure 12, to obtain the variation law of the dimensionless broken lengths of the jackets with impact velocity; it is evident that there exists an approximately linear and positive proportional relationship between them. The dimensionless broken lengths of the jackets in different scale models with the same impact velocity are approximately equal; i.e., they are independent of the geometric parameters of the projectile and target, which further indicates that the broken length of the jacket of the RIC-PELE after perforating the steel plate approximately follows the geometric similarity law. By using the fitting equation writing method in the previous text, through juxtaposed fitting, a straight line describing the relationship between the dimensionless broken length of the jacket and the impact velocity *v* is given as follows:

(27) $$\frac{L_{b}}{l}=0.42+(2.09\times 10^{-4})\frac{v}{v_{1}}$$

where *R^2^* = 0.98. Equation (27) is ultimately written as follows:

(28) $$\frac{L_{b}}{l}=0.42+0.209\frac{v}{v_{r}}$$

In Equation (28), the intercept 0.42 has no practical physical significance. The slope 0.209 reflects the linear enhancement effect of impact velocity on the broken length of the jacket. Its magnitude indicates the sensitivity of the dimensionless fragmentation length to changes in velocity.

As can be seen in Figure 12, the dimensionless broken lengths of the jackets of different scale models at the same speed do not completely overlap. Overall, the values corresponding to large-scale models are larger than those of small-scale models; however, the deviations among different scale models are not significant. Taking the model with a scaling ratio of *γ* = 1 as the reference benchmark, a ±7% deviation in the results is demonstrated by the shaded zone in Figure 12. It can be seen that the dimensionless values of other scaled models all fall within this area, indicating that compared with the results of the *γ* = 1 model, the absolute deviations of the dimensionless broken lengths of the jackets in other models are less than 7%. Generally, such deviations are acceptable. The primary reason for such deviations may be the scale effect induced by the strain rate effect of the materials. The strain rate is a function of velocity and penetrator size—the smaller model exhibits a higher strain rate, which enhances the dynamic strength of both the penetrator and the target plate simultaneously. However, under such circumstances, the scale effects generated in the penetrator and target plate could be approximately offset, thereby reducing the deviations in the dimensionless broken length of the jacket among models with different scaling ratios [58]. Consequently, the scale effect is insignificant and can be neglected, and the geometric similarity law for the broken length of the jacket is essentially valid.

### 4.4. Normalization of the Dispersion Radius of Jacket Fragments Behind the Steel Plate

**Figure 13:**
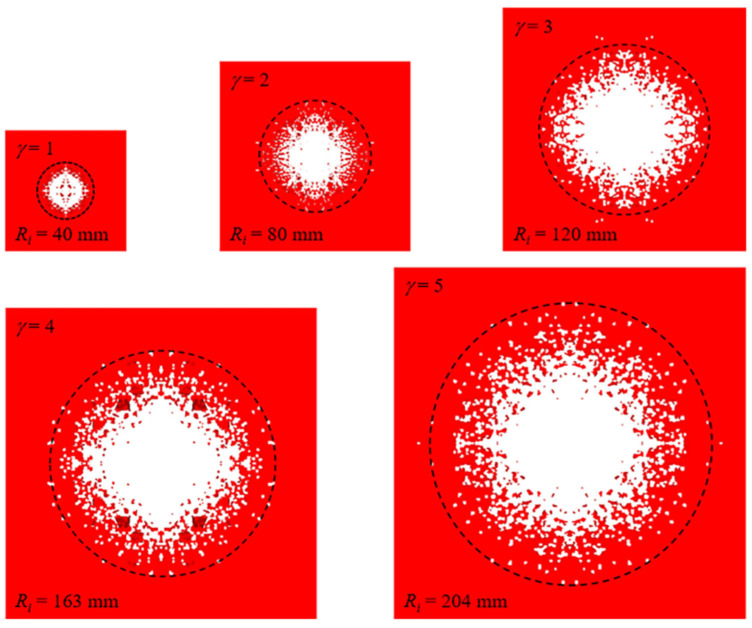
Distribution of jacket fragments from different scale models on the aluminium witness plates at an impact speed of 1500 m/s.

The distribution of RIC-PELE jacket fragments on the aluminum witness plate for the five scaled simulation models at an impact velocity of 1500 m/s is presented in Figure 13. Unlike the damage mode of the witness target shown in Figure 5, the witness targets in the scaled simulation models exhibit typical perforation damage, with many small perforations distributed around a large central perforation. The reason for this is that, in the scaled simulations, both the relative thickness of the steel plate (*H*/*R_j_*) and the relative distance between the front and rear targets (*D*/*R_j_*) are smaller, and the velocity of the penetrator is higher. After the penetrator perforates through the steel plate, the reaction degree of the reactive material and the radial scattering velocity of the jacket fragments are significantly different from those in the experimental simulations, resulting in differences in the damage modes of the witness targets. For the models with *γ* = 1, 2, 3, 4, and 5, the dispersion radii of the jacket fragments on the aluminum plate are 40, 80, 120, 163, and 204 mm, respectively, which approximately vary according to the scaling ratio of the models. This variation law is consistent with that of the geometrically similar traditional PELE [15].

**Figure 14:**
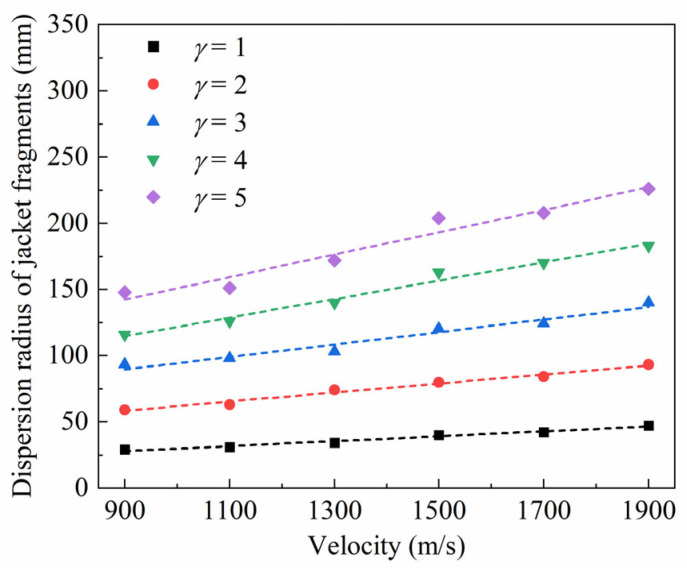
The variation in the dispersion radius of the jacket fragments with the impact velocity.

The variation in the dispersion radius of RIC-PELE jacket fragments on the aluminum witness plate with impact velocity for five scaling ratios is presented in Figure 14. For models with an identical scaling ratio, the dispersion radius of the jacket fragments behind the steel plate increases linearly with the rise in the impact velocity. As the velocity increases, the jacket fragmentation becomes more severe and the radial scattering velocity of jacket fragments behind the steel plate increases, which is the principal reason for the increase in the dispersion radius of the jacket fragments [8,10,59]. In addition, the higher the impact velocity, the more intense the chemical reaction of the reactive material, and the released gas products also make an important contribution to the radial scattering velocity of the jacket fragments [60]. At a constant impact velocity, the dispersion radius exhibits an increasing trend with larger scaling ratios.

**Figure 15:**
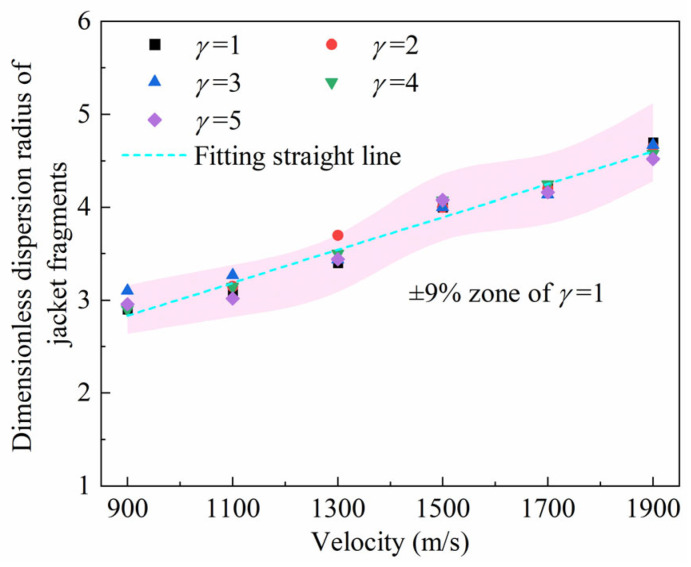
The variation in the dimensionless dispersion radius of the jacket fragments with the impact velocity.

The dispersion radius of the RIC-PELE jacket fragments in Figure 14 was normalized in accordance with the left term of Equation (19), and the variation law of the dimensionless dispersion radius of the jacket fragments behind the steel target with the impact velocity was obtained, as shown in Figure 15. The dimensionless dispersion radius of the jacket fragments is approximately linearly related to the impact velocity; the higher the velocity, the greater its value. When the impact velocity remains constant, the dimensionless dispersion radii of jackets fragments for different scale models are approximately equal and independent of the geometric parameters of the penetrator and target, indicating that the dispersion radius of RIC-PELE jacket fragments behind the steel plate approximately conforms to the geometrical similarity law. Referring to the classical similarity law, a straight line describing the relationship between the dimensionless dispersion radius of the jacket fragments and the impact velocity could be obtained by performing a juxtaposed fit, as shown in Equation (29):

(29) $$\frac{R_{i}}{d_{j}}=1.24+(1.77\times 10^{-3})\frac{v}{v_{1}}$$

where *R^2^* = 0.97. Equation (29) is ultimately written as follows:

(30) $$\frac{R_{i}}{d_{j}}=1.24+1.77\frac{v}{v_{r}}$$

In this equation, the intercept 1.24 also has no actual physical meaning. The slope 1.77 represents the linear enhancement effect of the impact velocity on the dispersion radius of fragments. The higher the velocity, the greater the lateral kinetic energy acquired by the fragments, and the larger the dispersion radius becomes. The magnitude of this coefficient reflects the sensitivity of the dimensionless dispersion radius to changes in velocity.

The ±9% deviation zone of the dimensionless dispersion radius of the jacket fragments with *γ* = 1 is the shaded area in Figure 15. Compared with this scale, the results of other scale models are within this deviation zone. This indicates that, for the dimensionless dispersion radius, the deviations existing between different scale models with an identical speed may also be caused by the scale effect resulting from the material strain rate effect. However, since the absolute values of the deviations are relatively small (i.e., less than 10%), they can be ignored. The results show that using the dimensionless dispersion radius of the jacket fragments of a small RIC-PELE to calculate this physical quantity of the large model proportionally results in a relatively high accuracy.

By analyzing the numerical simulation results, it was found that, ignoring the scale effect caused by the material strain rate effect, the broken length of the jacket and the dispersion radius of the jacket fragments behind the steel plate approximately obey the geometric similarity law when the RIC-PELE penetrates the steel plate. This verifies the correctness of the dimensional analysis on the similarity of the fragmentation effect of the RIC-PELE penetrating the steel plate.

In our research, the maximum magnification ratio of the model did not exceed five; therefore, the approximate geometric similarity law derived in this text is only valid under relatively small magnification ratios. When the model becomes too large, the influence of various factors cannot be ignored. Whether the broken length of the jacket and the dispersion radius of the jacket fragments approximately follow the geometric similarity law still requires further study. For the three fitting linear equations, their fitting coefficients are only applicable to the current material system. For other reactive core PELE configurations with different energetic compositions, the fitting coefficients will change. However, this linearly increasing trend might also be applicable to them.

## 5. Conclusions

In this paper, the broken length of the jacket and the dispersion radius of the jacket fragments behind the target were selected to measure the fragmentation effect of a RIC-PELE penetrating a steel plate. Through similarity analysis and numerical simulation, the following main conclusions could be drawn:

1. The dimensionless functions of the broken length of the jacket and the dispersion radius of jacket fragments behind the target, which were established via dimensional analysis, only take geometric dimensionless parameters as their independent variables.
2. Both the dimensionless broken length of the jacket and the dimensionless dispersion radius of the jacket fragments exhibit an approximate linear increase with increasing impact velocity, and their relationships with the impact velocity can both be expressed by linear equations. At the same velocity, for different scale models, the dimensionless broken lengths of the jackets are approximately equal, and the dimensionless dispersion radii of the jacket fragments behind the target are also approximately equal; compared with the reference benchmark model, the absolute deviations of these two dimensionless quantities of different scale models are within 7% and 9%, respectively. This indicates that the geometric similarity laws of the broken length of the jacket and dispersion radius of jacket fragments behind the target are approximately valid without considering the scale effect of the model.
3. The numerical simulation results show that the fragmentation effect of the RIC-PELE penetrating the steel plate roughly follows the geometric similarity law when the scale effect of the model is ignored. Meanwhile, the correctness of the dimensional analysis was also verified by the numerical simulations.
4. When the geometric similarity ratio is no more than five, the fragmentation effect of a large RIC-PELE can be approximately predicted through the scaled-down model.

## Figures and Tables

**Table 1:** Shock EOS and failure parameters of the 30CrMnSiNi2A steel, 2024 aluminum, and unactivated PTFE/Al.

Material	*ρ*/(g/cm^3^)	*Γ*	*c*_0_/(m/s)	*s*	*C_p_*/(J/(kg·K))	*σ_T_*/MPa
30CrMnSiNi2A Steel	7.86	2.17	4610	1.73	460	1200
2024 Aluminum	2.78	2.0	5330	1.34	890	720
Unactivated PTFE/Al	2.2	0.9	1450	2.25	–	240

Notes: 1. “*σ_T_*” represents the failure parameter of the material. 2. The value of *σ_T_* for the activated PTFE/Al is the same as that for the unactivated material.

**Table 2:** Main parameters of the powder burn model for the activated PTFE/Al.

Material	*K*/GPa	*G*/mm^−1^	*c*	*C*_1_/(m/s)	*C*_2_	*D*	*e*/(GJ/m^3^)
Activated PTFE/Al	58	60	0.667	500	0	1.868787	8.78

**Table 3:** Johnson–Cook parameters of the 30CrMnSiNi2A steel, 2024 aluminum, and unactivated PTFE/Al.

Material	*A*/MPa	*B*/MPa	*n*	*C*	*m*	*T_m_* */K*
30CrMnSiNi2A Steel	1529.65	471.747	0.123	0.011	0.785	1800
2024 Aluminum	218	546	0.355	0.038	3.73	775
Unactivated PTFE/Al	48	64.1	0.574	0.219	0.226	653

**Table 4:** Comparison of simulation results with experimental results [30].

Target Plate	Experiment			Simulation			Error (%)
	Damage Model	Damage Size (mm)	*R_i_*/*d_j_*	Damage Model	Damage Size (mm)	*R_i_*/*d_j_*	
Main Target	Perforation	50 × 52	–	Perforation	52 × 52	–	–
Aftereffect Target	Rupture; Perforation	360 × 290	6.0	Rupture; Perforation	340 × 250	5.7	5

Note: The last column represents the error between *R_i_*/*d_j_*.

**Table 5:** Geometric parameters of different scale models (unit: mm).

*γ*	*L*	*R_j_*	*l*	*R_f_*	*R_t_*	*H*	*D*	*e*
1	33	5	30	3	30	3	65	0.5
2	66	10	60	6	60	6	130	1.0
3	99	15	90	9	90	9	195	1.5
4	132	20	120	12	120	12	260	2.0
5	165	25	150	15	150	15	325	2.5

## Data Availability

The original contributions presented in this study are included in the article. Further inquiries can be directed to the corresponding author.
